# Risk assessment of PM_2.5_ to child residents in Brazilian Amazon region with biofuel production

**DOI:** 10.1186/1476-069X-11-64

**Published:** 2012-09-14

**Authors:** Beatriz Fátima Alves de Oliveira, Eliane Ignotti, Paulo Artaxo, Paulo Hilário do Nascimento Saldiva, Washington Leite Junger, Sandra Hacon

**Affiliations:** 1 Public Health and Environment Post-graduation, National School of Public Health at Oswaldo Cruz Foundation, Rio de Janeiro, Brazil; 2 Department of Nursing, State University of Mato Grosso, Mato Grosso, Brazil; 3 Institute of Public Health, Federal University of Mato Grosso, Mato Grosso, Brazil; 4 Institute of Physics, University of São Paulo, São Paulo, Brazil; 5 Laboratory of Experimental Air Pollution, University of São Paulo Medical School, São Paulo, Brazil; 6 Institute of Social Medicine, State University of Rio de Janeiro, Rio de Janeiro, Brazil

**Keywords:** Particulate matter, Biomass burning, Risk assessment, Health effects, Children, Adolescents and Brazilian Amazon

## Abstract

**Background:**

Exposure to fine fractions of particulate matter (PM_2.5_) is associated with increased hospital admissions and mortality for respiratory and cardiovascular disease in children and the elderly. This study aims to estimate the toxicological risk of PM_2.5_ from biomass burning in children and adolescents between the age of 6 and 14 in Tangará da Serra, a municipality of Subequatorial Brazilian Amazon.

**Methods:**

Risk assessment methodology was applied to estimate the risk quotient in two scenarios of exposure according to local seasonality. The potential dose of PM_2.5_ was estimated using the Monte Carlo simulation, stratifying the population by age, gender, asthma and Body Mass Index (BMI).

**Results:**

Male asthmatic children under the age of 8 at normal body rate had the highest risk quotient among the subgroups. The general potential average dose of PM_2.5_ was 1.95 μg/kg.day (95% CI: 1.62 – 2.27) during the dry scenario and 0.32 μg/kg.day (95% CI: 0.29 – 0.34) in the rainy scenario. During the dry season, children and adolescents showed a toxicological risk to PM_2.5_ of 2.07 μg/kg.day (95% CI: 1.85 – 2 .30).

**Conclusions:**

Children and adolescents living in the Subequatorial Brazilian Amazon region were exposed to high levels of PM_2.5_ resulting in toxicological risk for this multi-pollutant. The toxicological risk quotients of children in this region were comparable or higher to children living in metropolitan regions with PM_2.5_ air pollution above the recommended limits to human health.

## Background

Air pollution is one of the several environmental factors that is having a serious impact on human health and quality of life. Particulate matter (PM) air pollution, measuring less than 2.5 μm, has been the focus of international concern due to its diverse contribution to the global burden of disease. There have been more than 2,000 peer-reviewed studies published since 1997 linking it to strokes, various respiratory and cardiovascular problems and premature death. Unfortunately, the majority of the planet still resides in areas where the World Health Organization Air Quality Guidelines of 10 μg/m^3^ (annual) and 25 μg/m^3^ (24-hour period) is exceeded [1].

Global estimates of ambient pollution levels have relied heavily upon either econometric or transport models mostly due to the lack of ground-level measurements of air pollution, especially PM_2.5_, which have been unavailable for most of the planet [1]. Yet, the distinctive nature of the emission source and the atmospheric and weather conditions has been found to either reduce or exacerbate the effects of PM_2.5_. Studies are needed to evaluate and provide insight in high-risk areas as to the exposure and risk as many of the forms of air pollution are beyond the control of individuals and require policy at the national and international levels.

Developing countries, such as Brazil have several factors that are contributing to rising levels of PM_2.5_ and the increasing respiratory morbidity and mortality rates in certain regions, most notably in the Brazilian Amazon [2,3]. In the last 20 years, Brazil has had drastic changes in its land use, specifically in the Amazon region [4,5], where conversion of forests to pastures represents a long-term emission source of biomass burning for the region[6]. Another important source of biomass burning that is significantly rising is from sugarcane production in the surrounding areas [7-9], caused by the expansion of the clean fuel industry.

Currently, Brazil is the largest world producer and exporter of alcohol. However 75% of sugarcane harvesting in Brazil is done manually and utilizes pre-harvest burning [7]. In the Amazon region the sugarcane harvest occurs between April and October during the months of low precipitation when fires substantially deteriorate the air quality in the surrounding areas. An area referred to as the “arc of deforestation” resting within the Amazon region is known for its intense biomass burning and is the source of the increasing levels of air pollution in the Amazon ecosystem [10]. These areas have reached PM levels of up to 350–450 μg/m^3^[11].

In terms of morbidity and mortality, most epidemiological studies indicate that there is no PM_2.5_ threshold concentration that does not cause negative health effects and even minor exposure is known to pose an additional risk to those with existing heart, respiratory or other chronic diseases [12,13]. The resulting effects of these pollutant levels have serious implications on the more than 22 million people living in the Amazon region, especially the more vulnerable population subgroups such as children and the elderly [2,3,14].

In this study, we apply the Risk Assessment Methodology [15] to evaluate the intake and toxicological risk of PM_2.5_ in children and adolescents in areas of high biomass burning of the Subequatorial Brazilian Amazon. We build upon a recent review by Oliveira et al. (2011) [16] which evaluated the physical and chemical characteristics of air pollutants in areas of high biomass burning and fossil fuel combustion in Brazil and factored the threshold dose response as determined by the US EPA. Here, we focus on the last two steps of the risk assessment process by estimating potential dose and risk quotient in scenarios of exposure regarding the local seasonality. We hope to provide data and information about the exposure risk of PM_2.5_ for children and adolescents in a region of high risk due to biomass burning and to contribute in the discussion of regulatory policies both national and internationally.

## Methods

### Study design

This study is a risk assessment of PM_2.5_ in the Tangará da Serra region, an area of intense biomass burning located in the Subequatorial Brazilian Amazon. We estimated the intake and toxicological risk of PM_2.5_ in children and adolescents between the age of 6 and 14 who had participated in a panel study to assess the respiratory function in the municipality of Tangará da Serra during the year 2008. We applied the methodology of the United State Environmental Protection Agency [15] and Agency for Toxic Substances and Disease Register [17], which was adapted to measure the local exposure to PM_2.5_ in regions of elevated biomass burning. We utilized the ratio of PM_2.5_/PM_10_ to calculate the PM_2.5_ values from the real time PM_10_ values. The questionnaires were utilized to factor in the local characteristics of the population to have a more accurate assessment.

The exposure assessment and risk characterization was carried out with a semi-structured questionnaire conducted with the schoolchildren’s parents to obtain information regarding their family’s health, socioeconomic information and smoke exposure. Additionally, the International Study of Asthma and Allergies in Childhood (ISAAC) phase 1 questionnaire was used to identify children and adolescents asthmatic [18]. Internationally standardized and validated for use in Brazil [19], the ISAAC phase 1 questionnaire is composed of eight questions related to the occurrence and frequency of wheezing, dyspnea and coughing. Asthmatics were considered those children (6 – 12 years old) who scored 5 or more points and adolescents (13+ years) who totaled 6 or more on the questionnaire.

### Area and population of study

**Figure 1:**
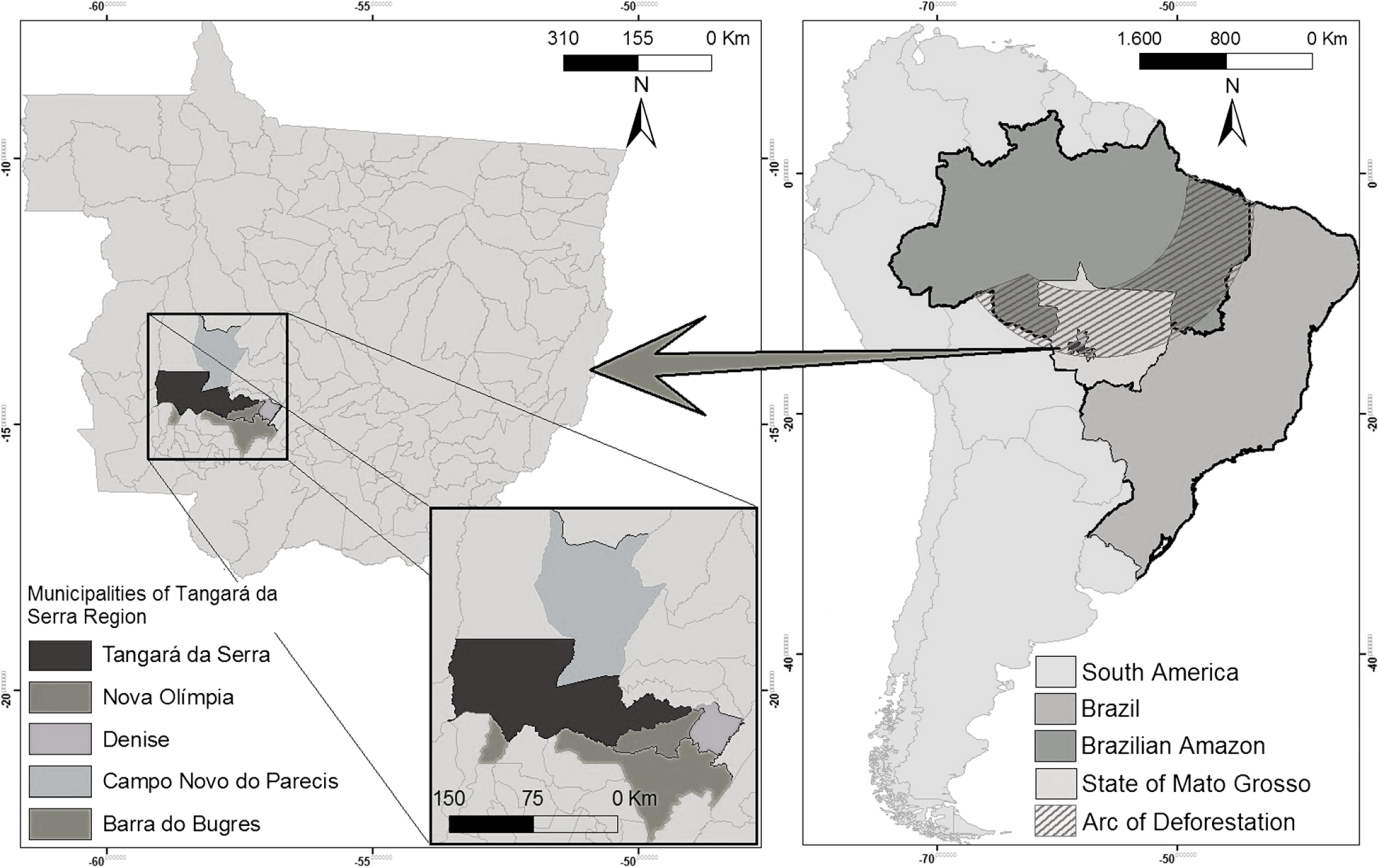
**Geographic region of Tangará da Serra and the other municipalities with the sugarcane plantations.** Suequatorial Brazilian Amazon, 2008.

This study was conducted in the municipality of Tangará da Serra situated at latitude 14° 37' 10'' to south and longitude 57° 29' 09'' to west of the Greenwich Meridian (See Figure 1). Located in the subequatorial Brazilian Amazon and the state of Mato Grosso, the *Tangará da Serra Region* has a large plume dispersion of pollutants coming from both neighboring countries as well as the arc of deforestation region. The territory stands out for PM emissions as a result of an increasing sugar cane plantation industry, where straw is burnt before the harvest. In 2008, the harvest was estimated to be 162,791 hectares, representing 60% the total cultivated area of the Mato Grosso State [20]. This municipality holds 50% of the region’s population, has an elevated prevalence of asthma [21] and high rates of hospital admissions for respiratory diseases [22].

The initial sample from pulmonary function study was 234 children. We included only those that responded to the semi-structured questionnaire. With a 95% response rate our study population consisted of a random sample of 221 children aged 6 to 14 (See Table 1). The students were from a public school that serves four districts in the area representing the diverse socio-demographic and health conditions of the city. Other than biomass burning, the school was not near any pollution source. This study was approved by Ethics Committee of the National School of Public Health (CEP/ENPS/FIOCRUZ - Protocol 75/10). Written informed consent was obtained from the parents of children for publication of this report and any accompanying images.

**Table 1:** Descriptive information of study participants

		**N**	**%**
**Age**	6 – 8	69	31.2
	9 – 11	69	31.2
	12 – 14	83	37.6
	**Total**	221	100.0
**Gender**	Male	100	45.2
	Female	121	54.8
	**Total**	221	100.0
**Asthma**	Yes	40	18.1
	No	181	81.9
	**Total**	221	100.0
**BMI**	Non-overweight	192	86.9
	Overweight	29	13.1
	**Total**	221	100.0

### Exposure scenarios

The risk assessment of PM_2.5_ was performed in two exposure scenarios, according to the seasonality of the region. The exposure scenarios were defined according to the average levels of PM and daily measure of relative humidity and precipitation. We defined two scenarios: July to October – the dry condition and November to December – rainy condition. In the Brazilian Amazon, the “dry season” is characterized by marked reduction in rainfall and humidity as well as an increased number of forest fires and high PM levels. The rainy period occurs between November and May when the precipitation and humidity reached average values of 10 mm/d (SD 17.7) and 83% (SD 8.3), respectively [23]. However, due to the lack of local infrastructure it was not possible to extend the rainy period beyond 60 days.

For both exposure scenarios, the probabilistic model was used to assess dose by the general equation of the potential dose. The input model variables and the assumed probability distributions were presented in Table 2. The mean dose of PM_2.5_ was calculated according to age groups, asthma status, gender and BMI (data not shown). To estimate the toxicological risk we calculated the risk quotient where the average dose of PM_2.5_ was related with the reference dose. The general equation of the potential dose and the toxicological risk to PM_2.5_ are described below [15]:

(1) Potential Dose of PM_2.5_

(1) $$\text{I}={\text{C}}_{\text{A}}\times \frac{\text{IP×FR×FA×ET×EF×ED}}{\text{BW}}\times \frac{1}{\text{AT}}$$

Where: The average daily concentrations of PM_2.5_ were measured at the air quality monitoring station of the University of Mato Grosso (UNEMAT/Tangara da Serra). This monitoring station is located in a radius of 5 km of the selected neighborhoods. Previous studies demonstrate that there is no significant difference in pollution levels within a 5-kilometer radius due to hotspots, plume dispersion and meteorological parameters [24]. The PM_10_ measurements were measured from the *Tapered Element Oscillating Monitor* (TEOM). The sampling device consisted of a stacked filter unit (SFU), which separates the aerosol into coarse and fine size fraction. The filter materials used were 8-μm pore size and 0.4 μm pore size polycarbonate filters for the coarse and fine size fractions, respectively. In this study, the daily concentrations of PM were equivalent to the daily ratio of PM_2.5_/PM_10_, which was applied, in the real-time measurements of PM_10_ (TEOM).

*I* Potential Dose (μg/kg.day)

*C*_*A*_ Concentration of PM_2.5_ (μg/m^3^)

*IP* Inhalation Rate (m^3^/day)

Inhalation rates were used in accordance with the EPA standards [25,26]. The probability gamma distribution was used to simulate the inhalation rate and body weight with the results of the adhesion Kolmogorov-Smirnov test placed in the best fit for the data.

*FR* Factor of Retention

This value was assumed as 1 representing the worst-case scenario and potential impact on people’s health.

*FA* Factor of Absorption

This value was assumed as 1 representing the worst-case scenario and potential impact on people’s health.

*ET* Exposure Time (hrs/day)

Exposure time was considered the amount of time in which the children were at the school – 4 to 8 hours/day. The children had at least 4 hours/day in the school having class (*the school conditions were without ventilation*). Moreover, we used the records from the panel study regarding the time each child spent in outdoor physical activity. These values were in accordance with the EPA standards [26]. This variable was placed in a uniform probability distribution adjusted for a minimum value of 4 hours/day and the maximum value of 8 hours/day.

*EF* Exposure Frequency (days/year)

The exposure frequency was the relative days in each PM_2.5_ exposure condition – corresponding to 76 days of dry scenario and 53 days of rainy scenario.

*ED* Exposure Duration (year)

July through December was defined as the exposure duration, portioning the corresponding 182 days into 122 and 60 days for the dry and rainy scenario, respectively. These variables remained constant in the model with no assumed probability distribution.

*BW* Body Weight (kg)

*AT* Average Time of Exposure (day)

July through December was defined as the exposure duration, portioning the corresponding 182 days into 122 and 60 days for the dry and rainy scenario, respectively. These variables remained constant in the model.

(2) Toxicological Risk

(2) $$RQ\;=\;\frac{I}{RfD}$$

Where:

*RQ* Risk Quotient

The Risk Quotient (RQ) estimated was appointed as RQ < 1 – Hazards that are not considered a threat to public health; and RQ > 1 – Hazards that cause the adverse health effects and are a detriment to public health.

*I* Potential Dose (μg/kg.day)

*RfD* Reference Dose (μg/kg.day)

With a lack of consensus regarding the Reference Concentration (R*f*C) of PM_2.5_, we used the 5 μg/m^3^ R*f*C of diesel particles (DPM) to calculate the reference dose to PM_2.5_ (R*f*D) to estimate the probability of adverse health effects. The DPM contribute a portion of ambient particulate matter and its reference dose has been established in the literature since 1993. To estimate the R*f*D, the average inhalation rate and body weight of all the children were used with only the extreme values of body weight (>77 kg) excluded. The extreme values of body weight represented 3% of the sample.

*RfC* Reference Concentration (μg/m^3^)

The inhalation R*f*C is calculated by the EPA with the Interim Methods for Development of Inhalation Reference Doses [15] and according to the Methods for Derivation of Inhalation Reference Concentrations and Application of Inhalation Dosimetry [27]. The diesel R*f*C, in particular, was calculated on 5 multi-dose studies [28-32], which provided the inter-study concentration-response continuum which was then normalized to human equivalent continuous diesel particulate matter (DPM) exposure levels and the R*f*C level was determined. The dosimetry model developed by Yu et al. (1991) [33] was used to calculate the human equivalent concentrations that corresponded to the animal values [34]. The EPA considers that the confidence level in the diesel R*f*C is considered medium in a range of low to high confidence.

**Table 2:** Description of input variables in the model to estimated the potential dose, according the exposure scenarios

**Input variables**	**N**	**Mean**	**SD**	**Minimum**	**Maximum**	**Distribution**
**Concentration** (**μg/m**^**3**^**)**						
Dry	76	41.9	31.1	5.9	130.0	Lognormal
Rainy	53	9.5	1.8	5.3	16.0	
**Inhalation Rate (m**^**3**^**/day)**						
Dry and Rainy	221	15.0	4.2	8.0	27.0	Gamma
**Body Weight (kg)**						
Dry and Rainy	221	36.0	13.4	17.0	82.0	Gamma
**Exposure Time (ET)**						
Dry and Rainy	-	-	-	0.16	0.33	Uniform
**Exposure Frequency (EF)**						
Dry	76					Constant
Rainy	56					
**Exposure Duration (ED)**						
Dry	0.67					Constant
Rainy	0.33					
**Average Time (AT)**						
Dry	122					Constant
Rainy	60					

### Statistical analysis

We combined random values from probability functions using a Monte Carlo simulation to estimate the most probable dose to which children were exposed in each exposure scenario. We estimated 200 doses of PM with the number of replications equal to 1000 resulting in a matrix. The potential dose is represented in the first column of this matrix. The results were log transformed and the difference of the PM mean doses between the groups was compared by *t*-Student Test and ANOVA at significance level of 5% (95% CI).

A sensitivity analysis was performed to verify the influence of the input variables in estimating the average dose of PM_2.5_. Spearman correlation coefficients were used to verify the influence of inhalation and body weight in the dose estimates – extreme weights were excluded. The rank correlation coefficients were squared and the values normalized to 100% to approximate the contribution to the variance*.* For the variables kept constant in the model (ET, EF, and DE) we increased these variables in 25% to evaluate their influence.

## Results

### Scenarios

The dry scenario (July-October) had a mean value of 56% relative humidity with 2.7 mm/d precipitation and average PM_2.5_ concentrations of 42 μg/m^3^ (SD 31 μg/m^3^). Whereas the rainy scenario (November-December) averaged 75% relative humidity with 5.3 mm/d precipitation and PM_2.5_ concentrations of 10 μg/m^3^ (SD 1.77 μg/m^3^).

### Potential dose of children (I)

#### Dry/Wet

Average potential dose of PM_2.5_ in the dry season was 1.95 μg/kg.day (95% CI: 1.62–2.27) and 0.32 μg/kg.day (95% CI: 0.29–0.34) in the rainy scenario (Table 3).

**Table 3:** **Potential dose of PM**_**2.5**_**incorporated according dry and rainy scenarios to age, gender, asthma and BMI**

	**Dry scenario (A)**			**Rainy scenario (B)**		
	**Mean**	**95%IC**		**Mean**	**95%IC**	
**Age**						
6 – 8	1.90*	1.71	2.08	0.33*	0.31	0.35
9 – 11	1.80	1.59	2.03	0.29*	0.27	0.31
12 – 14	1.67	1.47	1.86	0.26*	0.25	0.28
**Gender**						
Male	1.95	1.70	2.20	0.36	0.32	0.40
Female	1.96	1.71	2.21	0.34	0.31	0.38
**Asthma**						
Yes	2.11	1.86	2.36	0.32	0.29	0.35
No	2.06	1.81	2.31	0.30	0.27	0.32
**BMI**						
Non-overweight	1.74	1.53	1.95	0.33*	0.30	0.36
Overweight	1.58	1.40	1.76	0.27	0.25	0.29
**All children**	1.95	1.62	2.27	0.32	0.29	0.34

* ANOVA Test (p-valor <0.05).

#### Age

Children under the age of 8 had significantly higher (p-valor < 0.05) potential doses of PM_2.5_ than the adolescents between the ages of 12–14. The 6–8 year old children showed the highest potential dose of PM_2.5_ at 1.90 μg/kg.day (95% CI: 1.71–2.08) in the dry scenario (Table 3).

#### Asthma

Asthmatic children’s PM_2.5_ potential doses were 2.11 μg/kg.day (95% CI: 1.86–2.36) during the dry scenario. Non-asthmatic children’s PM_2.5_ potential doses were 2.06 μg/kg.day (95% CI: 1.81–2.31) dry scenario (Table 3). The PM dose of asthmatics was influenced by the individual characteristics of the children, such as the inhalation rate, body weight and biological vulnerability.

#### Weight

The approximately 13% of children considered overweight were exposed to 1.58 μg/kg.day (95% CI: 1.40–1.76) of PM_2.5_ during dry condition compared to children with a weight adequate for their age who incorporated 1.74 μg/kg.day (95% CI: 1.53–1.95) of PM_2.5_. No significant differences were observed between the mean doses incorporated between these two groups (Table 3).

### Toxicological risk (II)

The estimated reference dose (R*f*D) – was derived from the R*f*C of DPM (5 μg/m^3^) – proportioning 0.85 μg/kg.day in the dry condition and 0.60 μg/kg.day in the rainy condition.

**Figure 2:**
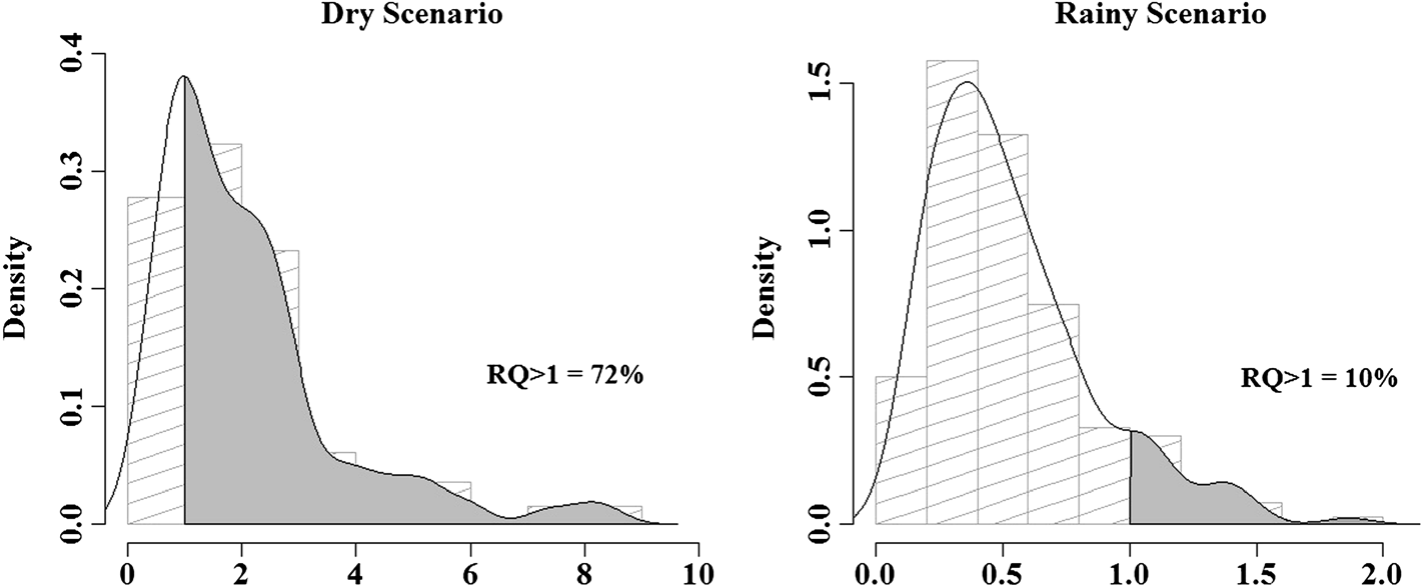
**Probability distribution of the toxicological risk to PM**_**2.5**_**for children during the dry and rainy scenario.** Subequatorial Brazilian Amazon, 2008.

Toxicological risk of PM_2.5_ was found for the dry condition (RQ = 2.07; 95% CI: 1.85–2.30). These results showed, that in the dry season, children were clearly exposed to levels of PM_2.5_ concentration that have the ability to cause adverse health effects. As expected, this result was not observed for the rainy condition (RQ = 0.45; 95% CI: 0.42–0.49) (Figure 2). In the dry scenario, the groups that showed the highest risks for potential adverse health effects were the children under 8 years of age and asthmatics. The toxicological risk was 2.23 (95% CI: 2.01–2.45) for children under the age of 8 and 2.48 (95% CI: 2.48–2.78) for asthmatics.

The probability distribution of toxicological risk was the lognormal distribution of the dry and rainy scenario (Figure 2). Approximately 72% of children aged 6 to 14 showed toxicological PM_2.5_ risk greater than 1 in the dry condition (Figure 2). Among the groups, 77% of children under 8 and 74% of asthmatics were exposed to a potential dose of PM_2.5_ capable to cause adverse health effects in the dry scenario.

### Sensitivity analysis

The exclusion of extreme weights to check the influence of inhalation rate and body weight increased the PM_2.5_ dose potential in 10% of the cases. The model for asthmatics was most sensitive with the exclusion of weight extremes reducing the PM_2.5_ dose potential by 13% and the variance by 26%. In evaluating the influence of exposure frequency and duration, the increase of 25% in these variables represented an increment of 31% in the doses of PM_2.5_. The variable used in the model that was most correlated with the PM_2.5_ dose was the concentration of PM_2.5_ (*r* = 0.83) in the dry scenario and body weight (*r* = −0.66) during the rainy scenario. The percentage that these variables contribute in the variance of the PM_2.5_ dose was by 70% and 44% in the dry and rainy condition, respectively.

## Discussion

A recent study by Bradshaw et al. (2010) [35] which ranked countries upon their relative and absolute environmental impact identified Brazil as having the highest contribution to global environmental degradation on the planet. The study which considered more than 170 different countries combined ranks for natural forest loss, habitat conversion, marine captures, fertilizer use, water pollution, carbon emissions and proportion of threatened species. Brazil was ranked having the most negative impact in natural forest loss on the planet, third in habitat conversion and fertilizer use, fourth in threatened species and carbon emissions and eighth in water pollution. It now surpasses countries such as the USA, China, Indonesia, Japan, Mexico, India and Russia as the country causing the most detrimental global environmental impact.

Much of this environmental impact is centered around the Brazilian Amazon region where this natural forest loss and habitat conversion has occurred. Guild et al. (2004) [6] identified that the forest loss in this region coupled with habitat conversion was producing a long-term emission source of biomass burning. Furthermore, with the increased awareness of the global environmental crisis there has been an increase in biofuel production, among which sugarcane is the fastest growing industry [9]. This expansion has driven Brazil to become the leading global biofuel producer and exporter of alcohol. Considered as a clean fuel, the burning of straw has caused serious concerns with public health in the regions surrounding sugarcane production [36]. Yevich and Logan (2003) [37] estimated that agricultural waste and biofuel burning is in a ratio of 50:50 in the case of sugarcane. This biomass burning has a significant impact on the global atmospheric chemistry; as such biomass energy is only partly renewable as its burning contributes to climate variability not to mention the air pollution in the regional area [4,38].

PM air pollution has been well documented to be hazardous to human health. Studies have found a strong exposure – response relationship between PM_2.5_ and both long and short-term effects that are further exacerbated in the ill, the elderly, or children [12,14,36,39]. That which is caused by biomass burning presents additional concern due to the environmental and atmospheric effects extending the exposure period to months rather than hours [14]. As well, the relative humidity and temperature play significant roles in exacerbating asthma and COPD [40], which identifies tropic regions as high risk areas. The dangers of air pollutants such as PM_2.5_ in developing countries such as Brazil, Mexico and India are often further exacerbated by compromised health conditions resulting from poverty and inadequate living conditions. In a study evaluating 1 year old children in Mexico city a 10 μg/m^3^ increase in PM_2.5_ values within 3 days was associated with almost a 7% excess of infant deaths [41], whereas Sheers et al. (2011) [42] found that risk levels of infant mortality due to PM levels increased by 4% among European children under the age of 1.

As expected, children between the ages of 6 and 14 were exposed to a higher PM_2.5_ dose in the dry season during the period of intense fires than during the rainy season. The potential dose in the dry season was strongly influenced by PM_2.5_ concentration levels which reached maximum values of 130 μg/m^3^ during the month of October.

The highest risk groups within children were male asthmatic children under the age of 8 at normal weight during the dry season. The children under the age of 8 showed toxicological PM_2.5_ risk 12% higher than the risk for adolescents (12–14 years). Our results corroborate other studies such as Calderon-Garciduanas et al. (2000) [43] which found that the long term inhalation of air pollutants such as PM was associated with lung hyperinflation indicative of small airway disease and that children between the ages of 7–13 had more symptoms when compared to 5–6 year olds and adolescents. Another study conducted in the metropolitan zone of Mexico city [44] utilized the same methodology of evaluating the health risk of PM_2.5_ inhalation, however, differentiated into three different age groups: children 2–6 years of age, children 6–12 years old and adults. The child groups were clearly higher than that of the adult group with the risk quotient of adults calculated at 1.15 where the 2–6 and the 6–12 year olds were 1.79 and 1.81, respectively. These studies concur with our findings regarding that the age range between 6 and 11 appears to have the highest level of exposure risk, most likely due to them breathing more per unit of body weight in this age strata. Furthermore, when comparing the Tangará da Serra region in the subequatorial Brazilian Amazon region with that of urban Mexico City the rates of risk are found to be comparable and in even some cases higher; 1.79 and 1.81 in Mexico City compared to 1.55 – 2.11 in Tangará de Serra region. It is important to note that in the Tangará de Serra region approximately 30% of children under the age of 8 were asthmatic and had the higher dose and risk of PM_2.5_ (2.11).

The different exposure circumstances of children make them a vulnerable population and are important to consider [26]. Their lungs are not fully developed and they have greater exposure than adults as they are outside more, as well, are more active [45]. Higher rates of mouth breathing and ventilation may draw pollutants even further into children’s lungs making them more difficult to remove [46]. Exposures in children can also deliver higher doses of different compositions that may remain in the lung for a greater duration [45].

Calculating risk assessment of air pollutants poses particular difficulties. In assessing risk, the particles exposed to the population must be identified and the danger and behavior of these substances in the human body must be assessed [15,26]. Moreover, calculating the dose reaching the target organ remains a challenge, while a theoretical exposure dose can be calculated from the air pollution concentration and exposure time, there still remain significant limitations in this approach.

In our exposure assessment process we attempted to diminish variability by matching the risk assessment methodology to a specific population of children. We utilized the school area as a specific point in the region to estimate a designated portion of time at that exposure level to attempt to reduce the spatial variability. Clearly, there is variation as to the PM_2.5_ levels in the outdoor region of the residences of our population that are unable to be accounted for, however to account for the “microenvironment” of the residences we adjusted the temporal variability to estimate 4 to 8 hours of time spent outdoors [26]. Of course, there remains several factors and long-term variability such as the use of wood burning appliances which we attempted to assess in the exposure to smoking questionnaire and even more short-term differences such as weekend activities. In attempt to control for some of the intra-individual variability we selected a population where various personal information had been gathered regarding body weight, asthmatic conditions and age to estimate as accurately as possible variations within the individuals. Utilizing this method we found clear differences within the children as to their exposure risk. In this study, the reduction in the variance due to the exclusion of extreme weight resulted in low sensitivity. It is estimated that differences of 20% had a small contribution to the uncertainties in risk estimates [47].

The uncertainties associated with the PM_2.5_ incorporated dose were smoothed with probabilistic models. The probabilistic models considered the limitations in relation to the lack of information about environmental and individual factors. By definition, the uncertainties analysis refers to the model-specification error and data values that are not known with precision due to limited observations or estimation error [15,26]. The uncertainties in the environmental models were reduced by the precision of the input variables. Nevertheless, uncertainties inherent in social and environmental factors, exposure (source characteristics, style and life habits, exposure time) and health outcome (toxicokinetic and toxicodymamic factors) still remain.

Limitations that we feel should be highlighted are difficulties in the environmental measures of real-time PM, a lack of consensus in the literature about PM R*f*C and R*f*D and inhalation rate measurements obtained by studies conducted in other countries. In relation to the R*f*C, we used the reference concentration of diesel particles. There is some debate as to whether particles from biomass burning are more, as, or less toxic to that of diesel. Studies have shown diesel particles have higher concentrations of polycyclic aromatic hydrocarbons (PAHs) and metals [16,48], however the EPA considers that the risks remain comparable. Several studies have demonstrated similar effects of exacerbated asthma and respiratory problems with high-level exposure of both diesel and ambient PM_2.5_[49-52]. This study is one of the first about risk assessment of PM_2.5_ in areas of intensive biomass burning and presents a methodology that could be highly applicable to estimate and compare levels of risk across emission source, location and populations.

In our study, where we calculated exposure risk, the difference between children who were asthmatic s and non-asthmatics showed no significant differences in exposure; however this is not to say the physiological effect of the same dose will not be different within the two groups. Our study does demonstrate that upon the calculation of the hazard quotient and risk exposure, that asthmatics were not exposed to a higher dose implying that a mechanism independent than exposure to PM_2.5_ is mediating the asthmatic condition. This does not negate the several studies showing an exacerbation of the asthmatic condition (emergency visits, hospitalizations, prevalence) in short and long term exposure of PM_2.5_; several studies in the literature demonstrate that PM_2.5_ does appear to aggravate the symptoms of asthma [49-55].

Within the last few years huge advances have occurred in understanding the mechanisms involved in PM on human health [56,57]. Unfortunately, the more that is understood the larger we see is the scope of consequences of PM air pollution on human health particularly that of fine and ultra fine particles [56,58]. Thousands of articles have been published ranging from fetal exposure, infant mortality, asthma, cardiovascular disease, premature death of the elderly, different emission sources; though as we begin to untangle the plethora of variables which interact in the risk of human health - finding models and rates that are able to be compared are difficult. With different emission sources, temperatures and humidity factors we have difficulty creating a clear overall picture of the risk posed to human health in the different regions of the planet.

With ground-level measurements of PM_2.5_ air pollution having been unavailable for most of the planet [1,59], these discrepancies in data leave estimation models of global PM_2.5_ air pollutant concentrations wholly disproportionate, weighted heavily in North America, Europe and East and South Asia [1]. As much as there is a complexity in estimating the intake doses, the Risk Assessment methodology was simple and a comprehensive tool with respect to the PM_2.5_ exposure from biomass burning. In public health, the exposure quantification and risk estimates provide resources for planning and formulation of protection health policies [58] where comparisons across emission sources and location can be evaluated and compared to find more accurate global assessments. These probabilistic models evaluate the variability of input variables in order to produce estimates of risk, whose proper distributions can be inferred in the underlying populations [26].

The results of this study illuminate the necessity for national legislation in Brazil to regulate more rigorously PM air pollution in high biomass burning areas during the dry season. Our study disagrees with Brauer et al. (2011) [1] which estimate long-term average ambient concentrations of fine particles (PM_2.5_) in the Latin American region will be decreasing due to declining biomass burning in these regions. We agree with the recent study by Tsao et al. (2012) [9] which indicated that PM_2.5_ levels are not reducing but on the contrary are increasing annually. Natural forest loss continues within the Amazon ecosystem, with some areas such as Mato Grosso increasing burnt area in the Amazon biome 3 times more than the proceeding year (2008–2009) [60]. With continued demands for cleaner fuel driving the demand for increased sugar cane production and no national regulation we speculate that biomass burning area will not be decreasing but on the contrary will be increasing. Long-term regulation has been set forth in the state of Sao Paulo, yet the sugarcane industry has spread to areas outside of regulatory control. Factoring in the rising temperatures, sustained habitat conversion and increased sugar cane production, we speculate that PM_2.5_ values will continue to rise. Admittedly, Brauer (2011) [1] identifies a lack of ground level data from the Latin American region, however, with emerging levels of PM_2.5_ in the scientific literature there is clearly a devastating effect of the human activities occurring in the Brazilian Amazon that not only affect the health [2,3,14] of those in the surrounding regions but have a potential global environmental impact that will be our legacy [4,30].

## Conclusions

Risk Assessment Methodology was sensitive in accommodating characteristics in specific groups and producing estimates of risk for respiratory adverse health effects. It identified specifically children younger than 8 as having more vulnerability to high levels of PM_2.5_ in the Amazon region. It furthermore provides a basis for comparison between other studies using the same methodology across emission sources, age and region.

## Abbreviations

PM, Particulate matter; BMI, Body mass index; PHAs, Polycyclic aromatic hydrocarbon; CI, Confidence interval; SD, Standard deviation.

## Competing interests

The authors declare they have no competing interests.

## Authors’ contributions

BFAO, EI and SH designed and implemented the PM_2.5_ Risk Assessment methodology; WLJ and BFAO the statistical and sensitivity analysis; PA contributed with the air pollution and meteorological data. PHNS provided the data of the panel study. All authors contributed to revising the final manuscript. All authors read and approved the final manuscript.
